# Decreased muscle strength is associated with proinflammatory cytokines but not testosterone levels in men with diabetes

**DOI:** 10.1590/1414-431X20187394

**Published:** 2018-07-23

**Authors:** J.P. Ferreira, A.M.O. Leal, F.A. Vasilceac, C.D. Sartor, I.C.N. Sacco, A.S. Soares, T.F. Salvini

**Affiliations:** 1 Laboratório de Plasticidade Muscular, Departamento de Fisioterapia, Universidade Federal de São Carlos, São Carlos, SP, Brasil; 2 Departamento de Medicina, Universidade Federal de São Carlos, São Carlos, SP, Brasil; 3 Departamento de Gerontologia, Universidade Federal de São Carlos, São Carlos, SP, Brasil; 4 Departamento de Fisioterapia, Fonoaudiologia e Terapia Ocupacional, Faculdade de Medicina, Universidade de São Paulo, São Paulo, SP, Brasil; 5 Departamento de Fisioterapia, Universidade Ibirapuera, São Paulo, SP, Brasil

**Keywords:** Diabetes, Testosterone, Muscle strength, Cytokines

## Abstract

The aim of this study was to compare muscle strength in male subjects with type 2 diabetes mellitus (DM2) with and without low plasma testosterone levels and assess the relationship between muscle strength, testosterone levels, and proinflammatory cytokines. Males (75) aged between 18 and 65 years were divided into 3 groups: control group that did not have diabetes and had a normal testosterone plasma level (>250 ng/dL), DnormalTT group that had DM2 with normal testosterone levels, and the DlowTT group that had DM2 and low plasma testosterone levels (<250 ng/dL). The age (means±SD) of the groups was 48.4±10, 52.6±7, and 54.6±7 years, respectively. Isokinetic concentric and isometric torque of knee flexors and extensors were analyzed by an isokinetic dynamometer. Plasma testosterone and proinflammatory cytokine levels were determined by chemiluminescence and ELISA, respectively. Glycemic control was analyzed by glycated hemoglobin (HbA1C). In general, concentric and isometric torques were lower and tumor necrosis factor (TNF)-α, interleukin (IL)-6, and IL-1β plasma levels were higher in the groups with diabetes than in controls. There was no correlation between testosterone level and knee torques or proinflammatory cytokines. Concentric and isometric knee flexion and extension torque were negatively correlated with TNF-α, IL-6, and HbA1C. IL-6 and TNF-α were positively correlated with HbA1C. The results of this study demonstrated that muscle strength was not associated with testosterone levels in men with DM2. Low muscle strength was associated with inflammatory markers and poor glycemic control.

## Introduction

Clinical and epidemiological evidence demonstrates that men with type 2 diabetes mellitus (DM2), metabolic syndrome, and obesity exhibit low plasma testosterone levels (1). Low testosterone levels are associated with metabolic and cardiovascular complications, sexual dysfunction, risk of bone fracture, and reduced muscle strength (2).

Around 20% of people with DM2 show a decline in testosterone levels (3) from disease onset (2). The causal interactions between obesity, metabolic syndrome, DM2, and testosterone deficiency are complex. In short, increased activity of the aromatase enzyme in adipose tissue raises estradiol levels, which inhibits the hypothalamic-pituitary-adrenal axis and prompts a decline in testicular production of testosterone. Additionally, hormones (leptin) and inflammatory mediators such as interleukin (IL)-1, IL-6, and tumor necrosis factor (TNF)-α in adipose tissue can also compromise testicular function (4).

Testosterone replacement therapy (TRT) can improve libido, sexual function, bone density, muscle mass preservation, body composition, mood, erythropoiesis, cognition, and quality of life as well as can lower the risk of cardiovascular disease (5) in obese men with DM2 (5,6). However, the topic is controversial because TRT is associated with increased risk of prostate cancer by worsening symptoms of benign prostatic hypertrophy, liver toxicity, hyperviscosity, erythrocytosis, severe heart failure, and cardiovascular disease, and exacerbates untreated sleep apnea, as previously reviewed (7–9). Thus, physicians should discuss symptom severity with the patients, including those resulting from loss of muscle strength, and weigh the potential risks and benefits of TRT (5,6).

Regardless of other factors, people with low testosterone levels develop reduced muscle strength (10). This is because testosterone stimulates protein synthesis and the recruitment of satellite cells (11). Additionally, testosterone inhibits production of the inflammatory cytokines IL-1 and IL-6 (12), which are known to activate apoptosis pathways and muscle atrophy (13). However, there is no study that analyzed the muscle strength of people with DM2 and the disease relationship to plasma testosterone levels. Although some research has shown that individuals with DM2 exhibit reduced muscle strength (14), whether low testosterone levels can exacerbate loss of strength in these subjects has yet to be established. Isokinetic dynamometry is a safe, reliable, and reproducible method to assess joint torque, providing the strength of individuals under different types of contraction (15). In addition, knee muscle strength plays an important role in movement and quality of life.

Therefore, this study aimed to evaluate the concentric and isometric torque of knee flexion and extension in diabetic men with and without hypogonadism. The presence of subclinical inflammation in DM2 and its influence on the musculoskeletal system is well known, activating pathways of apoptosis muscle atrophy (13) and reducing testicular testosterone secretion (16). As such, the present investigation analyzed participants' plasma levels of the proinflammatory cytokines IL-1, IL-6, and TNF-α.

Given that its association with subclinical inflammation means that DM2 can affect muscle strength (17), and that hypogonadism can also affect muscle strength (18) independently of DM2, the hypothesis of the study was that knee flexion and extension strength is lower in subjects with both DM2 and hypogonadism compared to subjects with DM2 and control subjects without hypogonadism.

## Material and Methods

### Subjects

**Figure 1.**
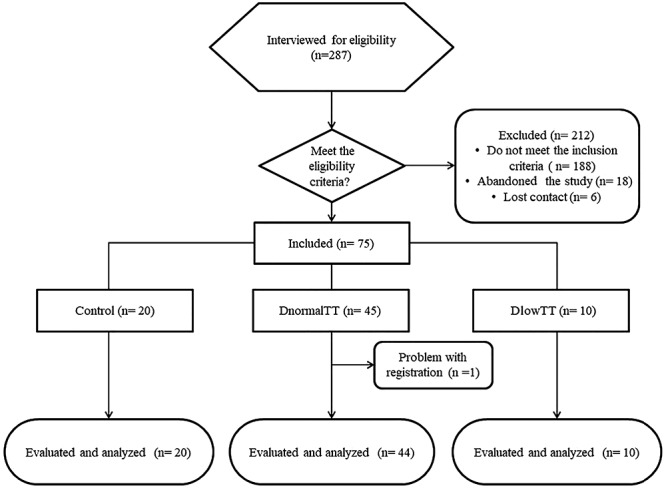
Flowchart of the study design. Control: control group; DnormalTT: diabetics with normal total testosterone; DlowTT: diabetics with low total testosterone.

The participants were recruited from local healthcare units and the Endocrinology Clinic of the Federal University of São Carlos. Males aged between 18 and 65 years were included in the study. The inclusion criteria for the control group were men without DM2 (19) and with normal total testosterone levels (20), for the DnormalTT group, men with DM2 (19) and normal total testosterone levels, and for the DlowTT group, men with DM2 (19) and low total testosterone levels (20). A total of 287 subjects were interviewed for eligibility. The exclusion criteria were cardiac diseases, pulmonary emphysema, knee arthrosis or arthritis, a history of knee ligament or meniscus injuries, herniated discs, stroke, peripheral diabetic neuropathy (PDN), and anti-inflammatory or hormonal therapy. The 75 subjects who met the inclusion criteria were distributed into the three groups: Control (n=20), DnormalTT (n=45), and DlowTT (n=10) (Figure 1).

The study complied with the Guidelines and Regulations for Research Involving Human Subjects (National Health Council Resolution 196/1996) and was approved by the university's Research Ethics committee (protocol No. 797.125). All participants took part voluntarily and gave written informed consent.

### Clinical evaluation

PDN was evaluated by a trained physical therapist using the following clinical parameters: i) typical neuropathy symptoms assessed by a questionnaire based on the Michigan Neuropathy Screening Instrument (21); ii) tactile sensitivity using a 10 g Semmes-Weinstein monofilament (Sorri-Baru, Brazil) tested in four plantar areas (hallux plantar face, and the 1st, 3rd, and 5th metatarsals); and iii) vibratory perception with a 128 Hz tuning fork applied to the medial region of the hallux interphalangeal joint (21). To determine the presence of PDN, these variables were processed in artificial intelligence Fuzzy Logic System software (LaBiMPH, Brazil), described in greater details in previous studies (22,23). The software combines each fuzzy set of the input variables and gives the degree of PDN between 0 and 10, classified as follows: i) <2.5 absent; ii) 2.5–5.0 mild; iii) 5.1-8.0 moderate; iv) >8.0 severe (22). The degree obtained by the fuzzy model showed a very strong correlation with the expert's assessment (Pearson's coefficient r=0.943) and a high level of accuracy when classifying real patients analyzed with the model (ROC curve area=0.91) (22).

### Anthropometric and glycemic control data

Body mass index (BMI) was calculated as follows: weight (kg) / height^2^ (cm). The glycated hemoglobin (HbA1c) percentage is a 3-month indicator of glucose control and was measured in all participants (19).

### Plasma testosterone

Total plasma testosterone level was determined by chemiluminescence (24). Data are reported in ng/dL and total testosterone <250 ng/dL was considered low (20).

### Proinflammatory cytokines

Plasma concentrations of TNF-α, IL-1β, and IL-6 were analyzed by sandwich ELISA (enzyme-linked immunosorbent assay) and each cytokine was tested according to the manufacturer's instructions (25). Readings were performed using a 490-nm filter. The detection limits of the cytokines in the serum were 5 pg/mL for IL-1β, 2.62 pg/mL for IL-6, and 1.7 pg/mL for TNF-α. After analyses, the data were transformed and normalized by the standard curve.

### Torque analysis

Concentric and isometric torque of knee flexors and extensors were analyzed using an isokinetic dynamometer (Biodex, System III, USA). Concentric torque was analyzed at 60°/s. The equipment was calibrated according to manufacturer's recommendations before the assessments. Individuals were asked which leg they could kick a ball hardest in order to determine their dominant limb. The type of contraction tested first was chosen randomly using a randomization spreadsheet. Before maximal testing, submaximal familiarization sessions were conducted with 3 repetitions for each movement in the concentric mode and 2 repetitions in the isometric mode. A 1.5-min rest was allowed between modes and 3 min between familiarization and the maximal test. During the test, participants remained seated in the dynamometer chair with the backrest at 85°, trunk stabilized and fixed to the backrest using two belts with an additional pelvic belt. The axis of rotation of the dynamometer was aligned with the lateral epicondyle of the femur and the resistance arm fixed to the distal third of the leg, just above the malleolus.

Verbal and visual encouragement was provided during all maximal voluntary contractions, always by the same evaluator. After the analyses, the text files of the tests were processed in MATLAB (version 7.0.1, MathWorks, USA) to determine the peak torque achieved by participants in the repetitions. Peak torque in N-m (newton meters) was normalized by the weight of the individual and multiplied by 100.

### Statistical analysis

The Levene and the Kolmogorov-Smirnov tests were applied to determine homogeneity of variance and normal distribution, respectively. One-way ANOVA and Tukey's post-hoc test were performed to compare the clinical and demographic characteristics of the groups, as well as concentric and isometric torque of the knee flexors and extensors. Significance was set at 5%. Effect sizes (Hedges’ g) of torque values between groups were also calculated, and considered insignificant (0.00–0.19), small (0.20–0.39), medium (0.40–0.79), or large (≥ 0.80) (26).

Kruskal-Wallis and Mann-Whitney U tests were applied to analyze the proinflammatory cytokines. For nonparametric comparisons, the significance level was adjusted according to the number of comparisons and set at P≤0.016. Correlations between concentric and isometric knee flexion and extension torque, proinflammatory cytokines, and HbA1c were assessed using Pearson's correlation coefficient (r=0.10–0.29: low correlation; r=0.30–0.49: medium correlation; r=0.50 –1: high correlation) (27).

## Results

One individual in the DnormalTT group was unable to complete the test. All groups were similar in terms of age. The DlowTT group had a higher BMI than the control group (P≤0.01); however, there was no difference in BMI between the two groups with DM2. Intergroup differences were observed for HbA1C values, but only between the groups with DM2 and the control group (P <0.01; Table 1).

**Table 1.** Clinical characteristics of the participants.

	Control (n=20)	DnormalTT (n=44)	DlowTT (n=10)	ANOVA
Age (years)	48.40 (10.03)	52.61 (7.81)	54.60 (7.24)	F=2.39; P=0.09
Time since diagnosis (months)	0.0 (0)	108.90 (72.48)*	89.40 (75.17)*	F=21.15; P=0.00
Testosterone (ng/dL)	402.0 (292.7)	369.2 (84.2)	204.6 (44.2)*^#^	F=0.60; P=0.55
HbA1C (%)	5.3 (0.4)	8.5 (2.5)*	9.1 (2.2)*	F=2.74; P=0.07
BMI (kg/m^2^)	26.4 (4.1)	27.8 (3.1)	30.7 (5.0)*	F=4.42; P=0.01
Degree of peripheral neuropathy (Fuzzy score)	0.67 (0.2)	1.2 (0.9)*	0.8 (0.2)	F=4.39; P=0.01
Oral antidiabetic / insulin / oral (n)		39/0/5	9/0/1	

Data are reported as means±SD. DnormalTT: type 2 diabetics with normal total testosterone; DlowTT: type 2 diabetics with low total testosterone. *P<0.05 compared to controls; ^#^P<0.05 compared to DnormalTT.

Peak torque was similar for all movements and both contraction types in the groups with DM2. When compared to controls, the DnormalTT group showed lower concentric and isometric knee extension torques (P<0.01) and the DlowTT exhibited lower isometric knee flexion torque (P=0.04). Both groups with DM2 showed lower concentric knee flexion torque than controls (P<0.02; Table 2).

**Table 2.** Peak torque for the different contraction types.

Type of Contraction	Joint movement	Control (n=20)	Effect size Control *vs* DnormalTT	DnormalTT (n=44)	Effect size DnormalTT *vs* DlowTT	DlowTT (n=10)	Effect size Control *vs* DlowTT	ANOVA
Concentric	Flexion	94.18 (33.87)	–1.58	53.35 (21.16)*	0.59	66.67 (28.37)*	0.85	F=16.8; P=0.00
	Extension	156.61 (56.50)	–1.17	104.36 (37.68)*	0.74	132.98 (41.79)	0.45	F=10.0; P=0.00
Isometric	Flexion	99.65 (35.77)	–0.65	80.46 (25.99)	-0.33	70.81 (40.73)*	0.77	F=3.73; P=0.02
	Extension	218.90 (54.04)	–0.81	171.95 (58.88)*	0.17	181.65 (43.10)	0.73	F=4.89; P=0.01

Peak torque is reported as means±SD (N.m/kg×100). DnormalTT: type 2 diabetics with normal total testosterone; DlowTT: type 2 diabetics with low total testosterone. Effect size: insignificant (0.00–0.19), small (0.20–039), medium (0.40–0.79), large (≥0.80). *P<0.05 compared to controls.

TNF-α, IL-6, and IL-1β concentrations were higher in the groups with DM2 than controls (P<0.01), but no difference was found between the DnormalTT and DlowTT groups (Table 3). HbA1c was positively correlated with IL-6 and TNF-α (r=0.55, P<0.01; r=0.59, P<0.01) and negatively correlated with concentric knee flexion and extension torque (r=–0.30, P<0.01; r=–0.24, P=0.03). Concentric and isometric knee flexion and extension torque were negatively correlated with TNF-α (r=–0.27, P<0.01; r=–0.33, P<0.01; r=–0.35, P<0.01; r=–0.43, P<0.01, respectively, for TNF-α *vs* concentric knee flexion; TNF-α *vs* concentric knee extension; TNF-α *vs* isometric knee flexion, and TNF-α *vs* isometric knee extension) and IL-6 (r=–0.22, P=0.03; r=–0.27, P<0.01; r=–0.38, P<0.01; r=–0.41, P<0.01, respectively, for IL-6 *vs* concentric knee flexion; IL-6 *vs* concentric knee extension; IL-6 *vs* isometric knee flexion, and IL-6 *vs* isometric knee extension). There was no correlation between testosterone level and knee torques, or proinflammatory cytokines, considering all groups.

**Table 3.** Comparison of inflammatory markers tumor necrosis factor (TNF-α), interleukin- 6 (IL-6), interleukin 1- beta (IL-1β).

	Control (n=20)	DnormalTT (n=44)	DlowTT (n=10)	Kruskal-Wallis test
TNFα (pg/mL)	0.71 (0.71–0.71)	0.74 (0.71–1.00)*	1.00 (0.74–1.00)*	H=46.2; P<0.01
IL-6 (pg/mL)	0.50 (0.51–0.51)	0.73 (0.51–0.83)*	0.73 (0.73–0.83)*	H=45.0; P<0.01
IL-1β (pg/mL)	0.87 (0.87–0.87)	0.90 (0.84–0.90)*	0.90 (0.84–0.90)*	H=14.0; P<0.01

Data are reported as means (minimum-maximum). DnormalTT: type 2 diabetics with normal total testosterone; DlowTT: type 2 diabetics with low total testosterone. *P<0.016 compared to controls.

## Discussion

The results of the present study showed reduced isometric and concentric torque in individuals with DM2 regardless of testosterone levels and their association with high IL-6 and TNF-α concentrations. These findings were consistent with the important pathophysiological role of inflammation in reducing muscle strength.

With respect to decreased muscle strength in individuals with DM2, our results confirmed the findings of previous studies showing that individuals with diabetes have lower skeletal muscle strength than those without diabetes (28,29). The mechanism of muscle strength decline in DM2 subjects is not well defined. In the present study, clinical neuropathy was excluded, but subclinical neuropathy could not be excluded.

We hypothesized that low testosterone levels would negatively influence decreased muscle strength in individuals with DM2. However, both diabetic groups (normal and low testosterone subgroup) exhibited lower muscle strength than control subjects and there was no correlation between testosterone levels and muscle strength. Testosterone administration has been associated with increased muscle strength. However, previous studies (10,30) that examined the correlation between endogenous testosterone and muscle strength have been inconclusive, possibly because circulating testosterone levels may not directly or linearly reflect its biological action on target tissues.

Mechanistically, the interaction between low muscle strength and high HbA1c may be explained by the effect of hyperglycemia on skeletal muscle mitochondrial dysfunction, protein degradation, and autophagy pathways (31,32), as well as the accumulation of advanced glycation products and oxidative stress (33).

Another potential mechanism for decreased muscle strength in individuals with DM2 is the rise in inflammatory cytokine levels. In the present study, the inflammatory markers TNF-α, IL-6, and IL-1β concentrations were higher in both groups with DM2 than in controls. It has been previously demonstrated that increased plasma concentration of inflammatory cytokines may cause loss of strength in these individuals (13,34). Inflammatory cytokines may lead to the inhibition of skeletal muscle protein synthesis and myoblast differentiation by activating MAFbx/atrogin-1, eIF3-f, MyoD, and MuRf1 (35–37 [Bibr B38]). In addition, it has been reported that increased TNF-α levels may activate the caspase pathway, leading to skeletal muscle atrophy due to enzymatic fragmentation of muscle DNA (38,33).

In conclusion, the results of this study demonstrated that muscle strength was lower in individuals with DM2 and was not associated with low total testosterone levels. Our results also indicated that reduced muscle strength in subjects with DM2 subjects could be associated with an increase in the inflammatory cytokines IL-6 and TNF-α and poor glycemic control. However, further studies are needed to clarify this association.

As limitations, the DlowTT group was a very small sample and despite the number of individuals interviewed, only 10 individuals with DM2 and low testosterone met the eligibility criteria of the study. Given the sample size evaluated in the DlowTT group, one-way ANOVA as the statistical design, and an alpha error of 5%, the statistical power (1 - β) obtained using a small effect size was 0.70. We also calculated the sample size using a power of 0.93 and medium effect size (0.45). For future studies, we recommend using a sample size of 75 individuals equally distributed among groups. In addition, it would be interesting for future studies to analyze the association between strength and low testosterone level with the quality of life and physical activity level of these individuals.
